# Imaging diagnosis for intervertebral disc

**DOI:** 10.1002/jsp2.1066

**Published:** 2019-09-29

**Authors:** Izaya Ogon, Tsuneo Takebayashi, Hiroyuki Takashima, Tomonori Morita, Yoshinori Terashima, Mitsunori Yoshimoto, Toshihiko Yamashita

**Affiliations:** ^1^ Department of Orthopaedic Surgery Sapporo Medical University School of Medicine Sapporo Japan; ^2^ Department of Orthopaedic Surgery Sapporo Maruyama Orthopaedic Hospital Sapporo Japan

**Keywords:** imaging diagnosis, intervertebral disc, magnetic resonance imaging

## Abstract

Various functional magnetic resonance imaging (MRI) techniques have been investigated in recent years and are being used in clinical practice for the patients with low back pain (LBP). MRI is an important modality for diagnosing intervertebral disc (IVD) degeneration. In recent years, there have been several reported attempts to use MRI T2 mapping and MRI T1ρ mapping to quantify lumbar disc degeneration. MRI T2 mapping involves digitizing water content, proteoglycan content, and collagen sequence breakdown as relaxation times (T2 values) at each site. These digitized values are used to create a map, that is, then used to quantitatively evaluate the metabolite concentrations within IVD tissues. MRI T2 mapping utilizes the T2 relaxation time to quantify moisture content and the collagen sequence breakdown. MRI T1ρ mapping digitizes water molecule dispersion within the cartilaginous matrix to evaluate the degree of cartilaginous degeneration. Magnetic resonance spectroscopy is a less‐invasive diagnostic test that provides biochemical information. Adequate analysis of the IVD has not yet been performed, although there are indications of a relationship between the adipose content of the multifidus muscle in the low back and LBP. The ultra short TE technique has been recently used to investigate lumbar cartilaginous endplates. Unlike diagnosis based on contrast‐enhanced images of the IVD, which depends on the recurrence of pain that is determined subjectively, MRI‐based diagnosis is less‐invasive and based on objective imaging findings. It is therefore expected to play a key role in the diagnostic imaging of IVD conditions in the future.

## Graphical abstract

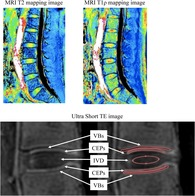

## 1 INTRODUCTION

Low back pain (LBP) is common disease, and is one of the most serious problems worldwide.^1^ LBP is classified into specific LBP, which is due to an obvious cause such as fracture, infection, or tumor, and nonspecific LBP, which is due to an unknown cause.^1^ Nonspecific LBP comprises 85% of all cases,^1^ and the intervertebral disc (IVD) was traditionally considered the site of origin for this type of pain.^2^ However, the underlying pathology remains unclear, and diagnosis and treatment are often difficult. In this article, we will summarize the latest information on diagnostic imaging for lumbar IVDs.

## 2 LBP AND IVD DEGENERATION

IVD injuries are caused by advancing age, mechanical overload, and genetic factors that all in turn result in degenerative changes. These injuries do not necessarily cause LBP,^3^ but it is important to differentiate physiological changes that occur as a result of advancing age from pathological changes that cause LBP.

### 2.1 The rationale for the IVD as the originating site of LBP

IVD‐related LBP is associated with abnormal findings on contrast‐enhanced imaging of the IVD. Because these changes resolve after lumbar vertebral fixation, it was generally believed that the IVD was the originating site of LBP. However, IVD degeneration is also sometimes observed in healthy individuals without LBP, so it has been impossible to conclude whether or not this condition is actually the root cause of LBP.

Sensory nerve fibers including mechanoreceptor nerve endings,^4^ small‐diameter myelinated nerve fibers, and unmyelinated nerve fibers^5^ are present in the outer layers of the annulus fibrosus. IVDs that exhibit degenerative changes express inflammatory cytokines and nerve growth factor (NGF). These substances have been shown to cause the nerve fibers that are normally only present in the outer layer of the annulus fibrosus to infiltrate the inner layer and the nucleus pulposus (NP) tissue,^6^ which is said to be the mechanism of IVD‐related LBP. In addition, the presence of sensory nerve fibers that contain neuropeptides related to inflammatory pain, such as substance P and calcitonin gene‐related peptide, has been confirmed in patients with LBP with features of IVD degeneration. Furthermore, clinical studies have reported a correlation between positive substance P test results in cases of IVD degeneration and the degree of LBP observed during contrast‐enhanced IVD imaging.^6^ IVD tissues in patients with LBP also contain an abundance of proinflammatory mediators compared to patients with lower limb pain alone, and these are reportedly expressed in even greater abundance in cases of IVD degeneration.^7^ It is therefore believed that inflammatory cytokines act on the nerve endings around the IVD to cause LBP. IVD injury‐related cytokines, such as interleukin (IL)‐1, IL‐6, tumor necrosis factor (TNF)‐α, and induced nitric oxide synthase are also observed in patients with back pain^8^, ^9^, ^10^ and are expressed in response to IVD ground substance hydrolysis products and mechanical overloading, thus promoting IVD degeneration. IVD degeneration causes damage to the outer layer of the annulus fibrosus and the cartilage endplate, which then activates repair mechanisms that form granulation tissue. This process causes simultaneous angioneogenesis, chondrocyte hyperplasia, and migration, which in turn results in conversion of the NP tissue into fibrocartilaginous tissue that further promotes IVD degeneration. In one sequence of the tissue repair process, NGF receptors (TrkA, p75), acid‐sensitive capsaicin receptors, namely, transient receptor potential V1, brain‐derived neurotrophic factor, and P2X3, an adenosine 5′‐triphosphate receptor involved in IVD metabolism, participate in the sensitization of sensory nerves in the degenerated IVD, and decrease the pain threshold for noxious stimuli. We believe that the information reported to date provides support for the idea that a painful IVD is the cause of LBP.

The cartilage endplate, like the annulus fibrosus, is supplied by the basivertebral nerve, which runs alongside the artery to the vertebral endplate and supplies the IVD tissues.^11^ The nerve density is similar to that in the annulus fibrosus, and clinical experience has shown that LBP is induced by the same mechanical stimuli as in the annulus fibrosus when lumbar vertebral surgery is performed under local anesthesia.^12^ Thus, it is thought that the cartilage endplate is one of the originating sites of LBP.

### 2.2 Diagnostic imaging of a painful IVD

It is extremely difficult to determine whether or not IVD degeneration is symptomatic based on imaging tests. One of the common physical findings of IVD‐related LBP is said to be pain intensification during anteflexion. However, in a study that investigated the preoperative clinical symptoms of patients with LBP who were diagnosed during contrast‐enhanced IVD imaging and improved after surgical fixation, pain intensification during anteflexion occurred in 65% of these cases, whereas pain intensification during retroflexion occurred in 35% of cases.^13^ Instability observed on dynamic plain X‐rays is also an indicator of IVD‐related LBP, although factors other than the IVD, such as the intervertebral joints or the interspinal and supraspinous ligaments, may be involved in intervertebral instability. IVD instability can therefore present in ways other than a painful IVD. Contrast‐enhanced imaging is commonly used to evaluate IVD‐related LBP. It is a valid method of evaluation with an approximate false positive rate of 10% in cases with no LBP, although the false positive rate increases to 40% to 80% in patients with a history of lumbar vertebral surgery, chronic LBP, and abnormal psychogenic reactions; consequently, some believe that the reliability of the site of pain onset as a specific indicator is low.^14^

Modic et al^15^ classified bone marrow changes in the vertebral endplate into three types in order to perform qualitative IVD diagnosis using magnetic resonance imaging (MRI). Type I, which presents with low intensity on T1‐weighted images and high intensity on T2 weighted images, is characterized by increased perfusion within the subchondral bone due to microinflammation; type II, which presents with high intensity on T1‐weighted images and high intensity on T2‐weighted images, is due to fatty degeneration of the bone marrow; and type III, which presents with low intensity on T1‐weighted images and low intensity on T2‐weighted images, is related to hardening of the subchondral bone. Studies have indicated that these findings are related to LBP, and a specific correlation has been observed between intervertebral instability and LBP when type I bone marrow changes are observed.^16^ Furthermore, cells that express the inflammatory cytokine TNF‐α are common in the cartilage endplates of patients with type I and type II changes who present with IVD‐related LBP.^17^ We believe that these findings are important to help identify painful IVD on MRI.

## 3 IVD AND ADVANCES IN DIAGNOSTIC IMAGING

Rapid advances in the qualitative diagnosis of IVD disorders using MRI have been made in recent years. In a normal IVD, the cartilage endplate is seen as an avascular field, so contrast medium is gradually dispersed into the IVD. In contrast, in IVD degeneration, radiating tears are present, so the contrast medium disperses instantaneously. In addition, the areas where the radiating tears are present are repaired by granulation tissue accompanied by neovascularization and nerve tissue, and these structures form bands on contrast‐enhanced MRI scans which demonstrates the usefulness of these images. Furthermore, a highly objective diagnosis can be made by simply quantifying the concentration of metabolites (such as water content and extra cellular matrix) within the IVD tissues, digitizing this content in each site in the IVD tissue, and then creating a map. This process has resulted in remarkable advances in the qualitative diagnosis of IVD conditions using MRI. Unlike diagnosis based on IVD contrast‐enhanced images, which depends on the recurrence of pain, that is, determined subjectively, MRI‐based diagnosis is less‐invasive and based on objective imaging findings and is thus expected to play a more central role in the diagnostic imaging of IVD conditions in the future.

Various functional MRI techniques have been investigated in recent years and are being used in clinical practice. MRI T2 mapping, which involves the digitization of water content, proteoglycan content, and collagen sequence breakdown as relaxation times (T2 values) at each site, enables the metabolite concentration within the IVD tissues to be quantitatively evaluated. In addition, MRI T1ρ mapping digitizes water molecule dispersion within the cartilaginous matrix, which enables the degree of cartilaginous degeneration to be evaluated. Magnetic resonance spectroscopy (MRS) is a less‐invasive test that provides biochemical information, and reports suggest a relationship between LBP and the adipose content of the multifidus muscle (Mm) in the lumbar region.

### 3.1 MRI T2 mapping

#### 3.1.1 Quantitative changes during IVD degeneration

**Figure 1:**
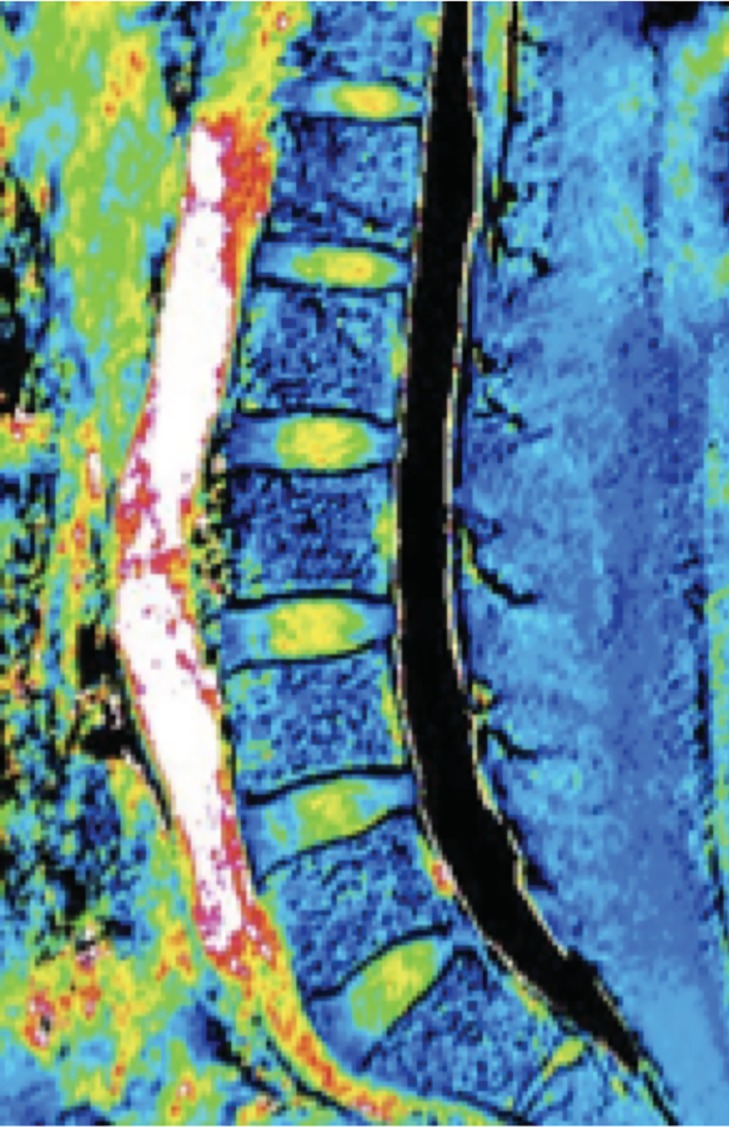
MRI T2 mapping image

**Figure 2:**
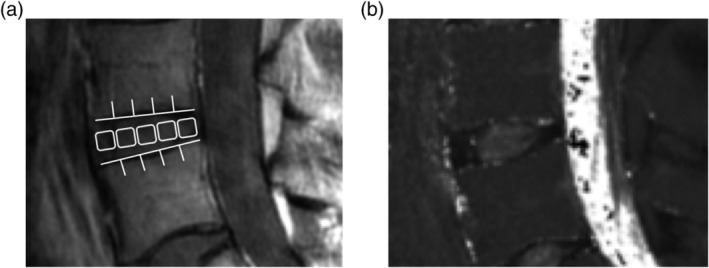
In second echo image, disc was divided into five areas, designating the front of the anterior annulus fibrosus (AF), the middle of the nucleus pulposus (NP), and the last of the posterior AF (A). In the same region, we measured the mean values (B)

**Figure 3:**
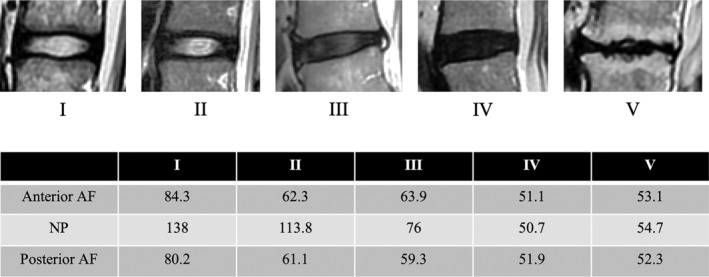
The case presentation of T2 values for each respective each Pfirrmann classification grade

IVD signal changes on T2‐weighted images reflect degeneration due to aging and allow the degree of IVD degeneration to be evaluated. Evaluation is possible because the signal intensity on MRI scans is related to water protons, and the proteoglycan and water content of the NP decreases in IVD degeneration. Pfirrmann et al^18^ created a classification system for the degree of IVD degeneration using MRI T2‐weighted images (Table 1); however, this system requires subjective visual evaluation, so a limitation in this regard is the absence of a quantitative method. We have quantified lumbar IVD degeneration based on the MRI T2 value using MRI T2 mapping (Figure 1). The disc was divided into five areas, designating the front of the anterior annulus fibrosus (AF), the middle of the NP, and the last of the posterior AF (Figure 2A). In the same region, we measured the mean values (Figure 2B). We analyzed the T2 values for each Pfirrmann classification grade (Figure 3) to conduct a comparative investigation, and receiver operating characteristic analysis was performed among grades to determine the cut‐off values.^19^ In the NP, T2 values tended to decrease with increasing grade, and there was a significant difference in T2 values between each grade from grades I to IV, but there was no significant difference between grade IV and V. T2 values according to disc degeneration level classification were as follows: grade I (>116.8 ms), grade II (92.7‐116.7 ms), grade III (72.1‐92.6 ms), and grade IV (<72.0 ms, Table 2).

**Table 1:** Classification of intervertebral disk degeneration as reported by Pfirrmann et al^18^

Grade	Structure	Distinction of nucleus and anulus	Signal intensity	Height of intervertebral disc
I	Homogeneous, bright white	Clear	Hyperintense, isointense to CSF	Normal
II	Inhomogeneous with or without horizontal bands	Clear	Hyperintense, isointense to CSF	Normal
III	Inhomogeneous, gray	Unclear	Intermediate	Normal to slightly decrease
IV	Inhomogeneous, gray to black	Lost	Intermediate to hypointense	Normal to moderately decreased
V	Inhomogeneous, black	Lost	Hypointense	Collapsed disc space

**Table 2:** Correlation with T2 relaxation time with intervertebral disc degeneration

	Grade I	Grade II	Grade III	Grade IV
NP	~116.8 ms	92.7‐116.7 ms	72.1‐92.6 ms	~72.0 ms

#### 3.1.2 Relationship between IVD MRI T2 values and LBP

We performed an analysis to determine whether there was a relationship between the MRI T2 value and scores on the lumbar visual analog scale (lumbar VAS) and the Japanese Orthopedic Association back pain evaluation questionnaire (JOABPEQ), using patients with chronic LBP as subjects. Our results showed a negative correlation between MRI T2 values for the posterior AF and the lumbar VAS and a positive correlation between these MRI T2 values and the JOABPEQ (pain‐related disorder) score.^20^ We also showed a correlation between posterior AF degeneration and neuropathic pain.^21^ The progression of IVD degeneration has been shown to be related to worsening of LBP and deterioration in quality of life. The area around the posterior AF is richly innervated by sinuvertebral nerves and sympathetic nerves derived from the nerve roots, and one mechanism that has been proposed is that these nerves penetrate into the deeper layers of the IVD.

#### 3.1.3 IVD MRI T2 values and LDS

Spinal column instability, which includes lumbar degenerative spondylolisthesis (LDS), is caused by degeneration of the IVD as the anterior support structure and the intervertebral joints as the posterior support structures. To clarify the details of IVD degeneration related to the onset of spondylolisthesis, we compared IVD MRI T2 values in patients with and without LDS. Our results showed that the anterior AF MRI T2 values were lower in patients with LDS than in those without LDS.^22^

### 3.2 MRI T1ρ mapping

**Figure 4:**
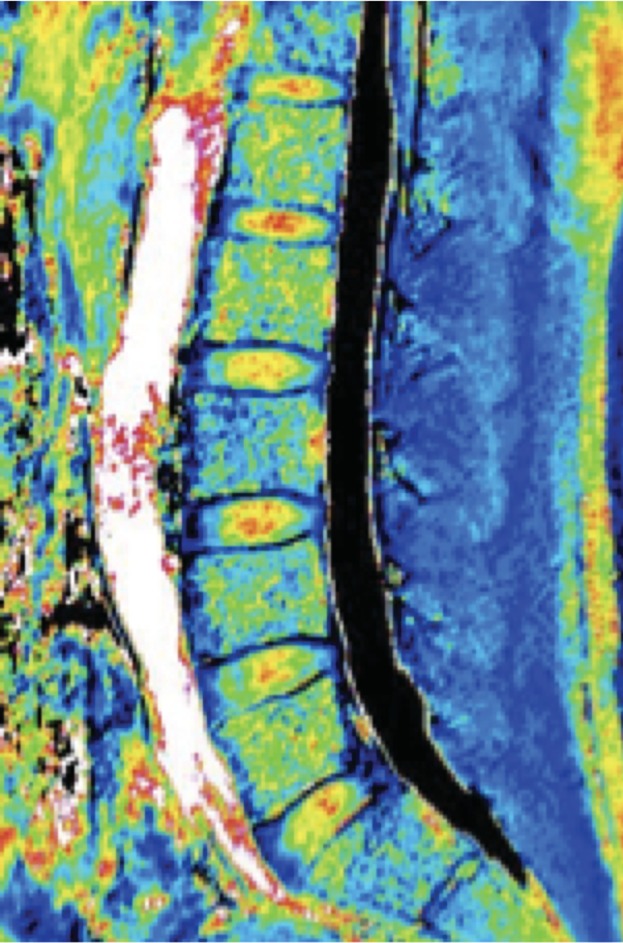
MRI T1ρ mapping image

T1ρ mapping digitizes water molecule dispersion within the cartilaginous matrix, which enables the degree of cartilaginous degeneration to be evaluated. Quantitative evaluation of IVD degeneration using this T1ρ mapping (Figure 4) is reportedly useful for diagnosing a painful IVD. Borthakur et al^23^ performed MRI T1ρ mapping in patients with LBP and reported that the T1ρ values decreased when the patients were suffering from IVD degeneration and that the IVD T1ρ values were lower in the group of patients with LBP than in the control group. In addition, IVD imaging in patients with LBP showed that the T1ρ value was lower in patients with a painful IVD than in those without IVD symptoms, and it is possible to perform MRI T1ρ mapping instead of invasive contrast‐enhanced imaging of the IVD to diagnose a painful IVD. Similarly, Blumenkrantz et al^24^ reported a correlation between the lumbar IVD T1ρ value and both the MOS 36‐Item Short Form Health Survey and the Oswestry Disability Index, so the T1ρ value has the potential to become an important indicator for evaluating LBP.

### 3.3 Magnetic resonance spectroscopy

#### 3.3.1 Relationship between the Mm fatty degeneration and LBP

The Mm contributes to two‐thirds of lumbar segmental stability and is said to play an important role in trunk function. We divided the fatty degeneration of the lumbar portion of the Mm into intramyocellular lipids (IMCL) and extramyocellular lipids (EMCL) components using MRS and found that IMCL was associated with chronic LBP.^25^, ^26^, ^27^

#### 3.3.2 Relationship between IVD MRI T2 values and the Mm fatty degeneration

The Mm and IVD are important for the function of the lumbar spinal column and degeneration in either impairs function. To clarify the details of IVD degeneration related to the fatty degeneration of the Mm, we compared IVD MRI T2 values IMCL and EMCL. Our results indicated that the IMCL of the Mm might be accompanied with posterior AF degeneration. The Mm as well as anterior AF of IVD were important for the function of the lumbar spinal column, and had cross‐correlation each other.^28^

Adequate analysis of the IVD using MRS has not yet been performed, although going forward, new methods of diagnostic imaging are expected to be applied to the IVD.

### 3.4 Ultra short TE

**Figure 5:**
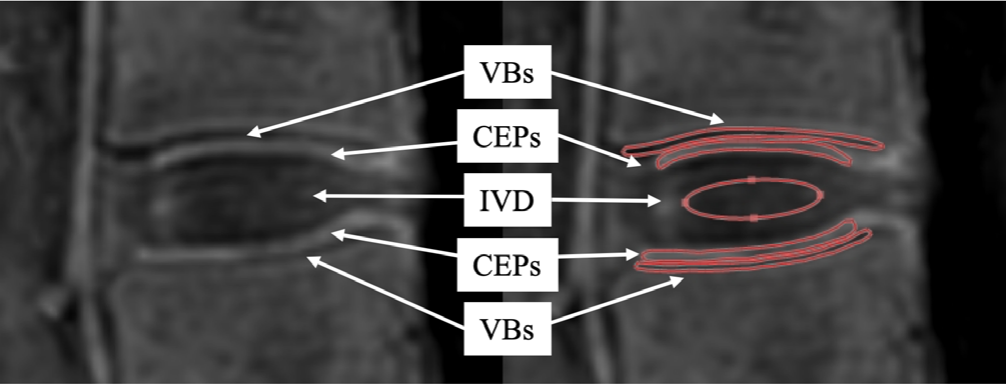
Regions of interests (ROIs) were positioned on cartilaginous endplates (CEPs), intervertebral disc (IVD), and vertebral bone (VB), and signal intensities (Sis) were measured. ROIs of IVD were located with the center of IVDs, and that of CEP, indicating for high signal between IVD and VB. Similarly, that of VB, a region of low signal adjacent to CEP

The ultra short TE (UTE) technique has been recently used to investigate lumbar cartilaginous endplates (CEPs).^29^ However, parameters of UTE have not been investigated, especially optimal second TE is unclear. We investigated the use of an optimal second TE with UTE for visualizing CEPs (Figure 5). A UTE sequence with fat suppression was used, and TEs were set at 0.16 ms as first TE, and 4.6, 9.2, 13.8, and 18.2 ms as second TE. Analyzed images subtracted each second TE image from first TE image. We showed the first TE was 0.16 ms, the optimal second TE was suggested to be 13.8 ms for evaluating human CEPs.^30^

## 4 CONCLUSION

Rapid advances in the qualitative diagnosis of IVD disorders using MRI have been made in recent years. Several reports have described the identification of the injury site using various imaging techniques and its relationship to pain. As an alternative to diagnosis based on IVD contrast‐enhanced images, which depends on the recurrence of pain, that is, expressed subjectively, MRI‐based diagnosis is less‐invasive and based on objective imaging findings, and it is therefore expected to play a more central role in the diagnostic imaging of IVD conditions in the future.

## CONFLICT OF INTEREST

The authors declare no potential conflict of interests.

## AUTHOR CONTRIBUTIONS

All authors contributed equally to the data research, discussion, writing, and revising of the manuscript.
